# Longitudinal study on the effect of keratinized mucosal augmentation surrounding dental implants in preventing peri-implant bone loss

**DOI:** 10.7717/peerj.13598

**Published:** 2022-06-28

**Authors:** Takeshi Kikuchi, Masahiro Wada, Tomoaki Mameno, Daisuke Hasegawa, Giovanni Serino, Kazunori Ikebe

**Affiliations:** 1 Department of Periodontology, School of Dentistry, Aichi Gakuin University, Nagoya City, Aichi, Japan; 2 Department of Prosthodontics, Gerodontology and Oral Rehabilitation, Osaka University Graduate School of Dentistry, Suita, Osaka, Japan; 3 Department of Periodontology, Södra Älvsborg Hospital, Brämhultsvägen, Borås, Sweden

**Keywords:** Dental implant, Keratinized mucosal augmentation, Longitudinal study, Peri-implant disease

## Abstract

**Background:**

Dental implant therapy is a well-established method of prosthetic rehabilitation of missing teeth. To maintain the health of the surrounding tissue, management of risk factors/indicators and daily maintenance are important. It still remains controversial whether a certain amount of keratinized mucosal width is essential for maintaining the health of peri-implant tissue. The purpose of this multicenter retrospective study was to assess the correlation between bone loss around dental implant and the amount of keratinized tissue width.

**Methods:**

A total of 1,644 implants were evaluated. Data was collected about participants’ general and dental history, as well as implant details. Bone resorption around implant was calculated from intra-oral radiographs taken after 1 year and more than 3 years of function. Implants were classified into three groups; received free gingival graft or apically repositioned flap surgery for increasing the keratinized mucosa ≥2 mm width (group A), keratinized mucosa width ≥2 mm (group B), and keratinized mucosa width <2 mm (group C). These data were analyzed by propensity score analysis and a generalized linear regression analysis was performed to compare the bone resorption among groups.

**Results:**

Mean functional time was 55.8 months (SD = 20.5) in group A, 67.6 months (SD = 28.1) in group B, and 74.5 months (SD = 32.9) in group C. Mean bone resorption of groups A, B, and C were 0.08 mm (SD = 0.40), 0.18 mm (SD = 0.66), and 0.44 mm (SD = 0.40). Groups A and B had significantly lower bone resorption than group C.

**Conclusion:**

The results in this study show the importance of keratinized mucosa in maintaining the peri-implant bone. Our findings also suggest that mucosal transplantation is useful, as opposed to narrowing of the keratinized mucosa.

## Introduction

Dental implant therapy is a well-established method to rehabilitate missing teeth and is highly beneficial for the recovery of masticatory function (Esposito et al., 2015). However, peri-implant diseases, a complication, occur at a constant rate. Peri-implant diseases are divided to two pathological situations. Peri-implant mucositis is defined as the presence of a plaque-related inflammatory soft tissue infiltrate with-out concurrent loss of peri-implant bone tissue, while peri-implantitis demonstrate inflammation in combination with bone loss. Peri-implant mucositis is reversible, meanwhile peri-implantitis is irreversible. Therefore, importance of prevention of peri-implantitis was highlighted, as mucositis was found to be potentially progressing into peri-implantitis. To maintain the health of the surrounding tissue, management of risk factors/indicators and daily maintenance are important. In a retrospective longitudinal study, poor oral hygiene, loss of occlusal support, maxillary implant site, cement-supported superstructure, and width of keratinized mucosa were risk indicators for peri-implantitis (Mameno et al., 2020). Many studies have suggested a prominent association between periodontitis-related factors such as a history of periodontitis and poor oral hygiene and peri-implantitis (Renvert & Quirynen, 2015; Schwarz et al., 2018). However, just as peri-implant tissue and the periodontium are deceptively similar, peri-implantitis and periodontitis have different basic properties while having common characteristics (Kotsakis & Olmedo, 2021).

It is commonly believed that width of keratinized and attached gingival width is not important in the progression of periodontal disease if good oral hygiene is maintained (Kennedy et al., 1985). Many studies have focused on the significance of keratinized mucosa surrounding dental implants for peri-implant health (Wennstrom & Derks, 2012; Lin, Chan & Wang, 2013; Thoma, Muhlemann & Jung, 2014). The authors also had previously reported a link between peri-implantitis and width of keratinized mucosa (Mameno et al., 2020; Wada et al., 2019). A systematic review found that an appropriate amount of keratinized mucosa is required to maintain the health of the tissue surrounding the implant (Pranskunas et al., 2016).

It has been reported that an insufficient amount of keratinized mucosa, up to 2-mm, tends to cause discomfort during brushing, plaque accumulation, and inflammation of the tissue surrounding the implant (Souza et al., 2016). The width of the keratinized mucosa on the buccal side of the implant is generally about 1 mm shorter than the width on the palatal/lingual side of the tooth (Chang et al., 1999; Chang & Wennstrom, 2013; Parpaiola et al., 2015). In contrast, it has been reported that the thickness of the buccal keratinized mucosa surrounding the implant is about 1 mm less in thickness than the gingiva (Chang et al., 1999). Considering the aforementioned reports and some contradictory findings (Kim et al., 2009; Schrott et al., 2009), it remains controversial whether a certain amount of keratinized mucosal width is essential for maintaining the health of peri-implant tissue.

To clarify the risk factors of bone resorption around a dental implant, appropriate statistical analysis is crucial. Propensity score (PS) analysis is a useful statistical technique to adjust for confounding factors. However, few studies have investigated the necessity of keratinized mucosa using PS analysis.

The purpose of this multicenter retrospective study was to clarify the correlation between the presence of keratinized mucosa and peri-implant bone resorption and the clinical efficacy of augmentation techniques used to increase the keratinized mucosal width around dental implants by PS analysis.

## Materials & Methods

### Study participants

The records of patients who underwent dental implant therapy between November 1996 and February 2015 at two university dental hospitals (Osaka University Dental Hospital, Osaka, Japan and Aichi Gakuin University Dental Hospital, Aichi, Japan) and six dental offices, which also situated in Japan were analyzed. The inclusion criteria were: (1) at least one rough-surface titanium implant with fixed prosthesis in function for over 4 years and availability of intraoral radiographs taken at 1 year follow-up after prosthesis delivery. The exclusion criteria were: (1) absence of the regular maintenance programs, (2) history of radiotherapy to the head/neck area, and (3) presence of uncontrolled systemic diseases. All participants provided informed consent after understanding the purpose of the study. Before implant therapy, all participants received initial periodontal treatment and smoking cessation guidance, if necessary.

This study was approved by the Osaka University Graduate School of Dentistry Ethics Committee (H28-E24). All clinical investigations were conducted according to the principles of the Helsinki Declaration. This study also followed the Strengthening the Reporting of Observational Studies in Epidemiology (STROBE) guidelines.

### Demographic data collection

Data on age, sex, smoking habits (defined as smoking more than one cigarette per day), alcohol consumption habits (defined by daily intake), and systemic diseases were obtained as systemic factors. As intraoral factors, history and presence of periodontitis, oral hygiene status (plaque control record (PCR); O’Leary score), number of occlusal support (Eichner index; A1–3, B1–4, and C1–3), existence of bruxism, and gonial-angle on the orthopantomogram (index of occlusal force) were collected (Miwa et al., 2019). The definition of periodontitis is currently followed a new classification, which based on a multi-dimensional staging and grading categories (Papapanou et al., 2018). However, the data of this study was collected retrospectively, therefore, definition of periodontitis in this study referred to the previous report (Derks et al., 2016). According to this report, history of periodontitis was defined as recorded presence of bleeding on probing or suppuration, attachment loss ≥ 2 mm, and pocket probing depth ≥ 2 mm on more than two teeth, and presence of periodontitis was defined as bleeding on probing or suppuration attachment loss ≥ 2 mm, and pocket probing depth ≥ 6 mm on more than two teeth at the follow-up examination, according to a previous report of periodontitis definition. Based on these data, the participants were divided into two groups by using a cut-off value of 20% PCR score, and Eichner A1–3, B1–2, B3–4 (having at least one occlusal contact), or C1–3 (no occlusal contact). Bruxism was diagnosed if the following signs were present: subjective symptoms of tooth grinding or clenching, abnormal tooth wear, and transient pain or fatigue of the masseter muscle.

### Implant data collection

Functional time, implant length and diameter, arch (maxillary or mandibular implant), implant site (anterior or posterior, distal to the canine tooth was defined as posterior), surgical procedure (one-stage or two-stage), with/without bone augmentation (guided bone regeneration (GBR), sinus lift, and socket lift), with or without free gingival graft (FGG) or apically repositioned flap surgery (APF), connection type (external or internal), fixation method (cement-retained or screw-retained), and keratinized mucosa width (KMW). KMW was defined as the minimum distance between the gingival margin and the mucogingival junction surrounding the implant. APF was judged necessary when the KMW was between 1 mm and 2 mm, and FGG was judged necessary when the KMW was less than 1 mm. In practice, APF and FGG were performed with the consent of the participants. Based on this, implants were categorized as follows: received FGG or APF (group A), KMW ≥ 2 mm (group B), and KMW < 2 mm (group C).

### Radiographic evaluation

In this study, bone resorption around each implant was evaluated by intraoral radiography. A single blinded examiner (MW) analyzed the bone level using image analysis software (ImageJ 1.49v; Wayne Rasband, National Institutes of Health, Bethesda, MD, USA). The measurement method was as follows: the vertical distance from the implant apex to the bone crest in contact with the implant was measured on the mesial and distal to the implant. The actual implant length was then used to calibrate the vertical distance. This measurement was performed at baseline (1 year after prosthesis delivery) and at follow-up (over 3 years from baseline). The marginal bone level change (MBLC) was calculated by the difference in the distance between baseline and follow-up. If there was a difference between mesial and distal MBLC, a larger MBLC was used for analysis. As bone resorption within the first year of implant function is often relatively high because of bone remodeling, the baseline was set to 1 year after delivery of the prosthesis, which is considered to be the period of completion of physiological bone resorption. It should be noted that the intra-class correlation coefficient for the MBLC measurement was 0.97, which indicated almost perfect concordance.

### Statistical analysis

The mean and standard deviation (SD) for continuous variables and percentage for categorical variables are presented as descriptive statistics. Initially, the comparison analyses for each variable were performed by Chi square test and Kruskal–Wallis test. Inverse probability of treatment weighting (IPTW) was used to address the large differences in sample size and baseline characteristics of the study population. In the IPTW method, weights are assigned to implant sites based on the inverse of their probability of receiving a keratinized mucosal augmentation procedure, as estimated by the propensity score (PS) analysis. The score was calculated based on a multinomial logistic regression model that estimated treatment allocation to the three groups, along with background factors associated with bone resorption (age, sex, follow-up period, history of periodontitis, diabetes mellitus, smoking, PCR) as adjusting variables. IPTW results in a pseudo-population in which implant sites with a high probability of receiving the procedure have a smaller weight and implant sites with a low probability of receiving the procedure have a larger weight. Therefore, the distribution of the measured characteristics of implant sites that are used to calculate the propensity score becomes independent of the treatment assignment. Finally, a generalized linear regression analysis was performed to compare the MBLC among groups A, B, and C in the re-weighted pseudo-population. All statistical analyses were performed using R version 3.31 (The R Foundation for Statistical Computing, Vienna, Austria). The level of statistical significance for all analyses was set at 0.05.

## Results

**Figure 1:**
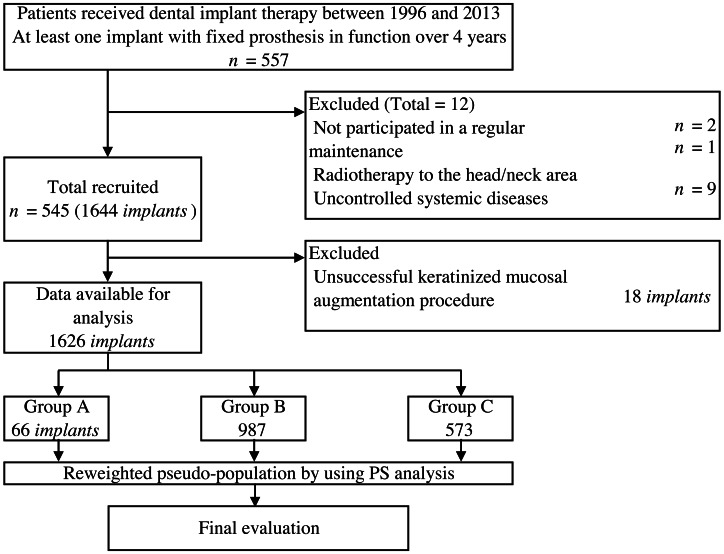
Flow of participants selection and analysis method.

A total of 1,626 implants (66 in group A, 987 in group B, and 573 in group C) were placed in 545 patients with a mean age of 57.5 years (Fig. 1). In group A, 18 implants (21.4%) with less than 2 mm KMW at follow-up were determined to be unsuccessful and excluded from the analysis. The description of all variables according to each group is presented in Table 1. Mean functional time was 55.8 months (SD = 20.5) in group A, 67.6 months (SD = 28.1) in group B, and 74.5 months (SD = 32.9) in group C. Mean MBLC of group A, B, and C were 0.08 mm (SD = 0.40), 0.18 mm (SD = 0.66), and 0.44 mm (SD = 0.40), respectively. Descriptive analysis showed large variations in baseline characteristics among groups A, B, and C.

**Table 1:** Characteristics of evaluated implants.

Categorical variables		Group A		Group B		Group C		*P* value	Total	
		n	%	n	%	n	%		n	%
Total		66		987		573			1626	
Sex	Men	26	39.4	366	37.1	209	36.5	.950^a^	601	37
	Women	40	69.6	621	62.9	364	63.5		1025	63
History of periodontitis	No	42	63.6	446	45.2	267	46.6	<.001^a^	755	46.4
	Yes	24	36.4	541	54.8	306	53.4		871	53.6
Implant position	Mandible	52	78.8	509	51.6	364	63.5	<.001^a^	925	56.9
	Maxilla	14	21.2	478	48.4	209	36.5		701	43.1
Fixation method	Screw	12	18.2	270	27.4	188	32.8	<.001^a^	469	28.8
	Cement	54	81.8	717	72.6	385	67.2		1157	71.2
Diabetes	No	66	100	919	93.1	529	92.3	.379^a^	1514	93.1
	Yes	0	0	68	6.9	44	7.7		112	6.9
Eichner index	A1-3	3	4.55	210	21.3	55	9.6	<.001^a^	268	16.5
	B1-4	60	90.9	673	68.2	391	68.2		1124	69.1
	C1-3	3	4.55	104	10.5	127	22.2		234	14.4
Continuous variables	Mean	SD	Mean	SD	Mean	SD		Mean	SD
Age	(Years)	55.8	7.3	59.0	10.8	62.3	9.5	<.001^b^	60.0	10.4
PCR	(%)	18.6	14.0	21.9	17.1	25.6	21.3	.071^b^	23.0	18.7
Smoking	(Cigarettes)	0.5	2.1	2.3	7.2	1.8	5.6	.061^b^	2.1	6.5
Functional time	(Months)	55.8	20.5	67.6	28.1	74.5	32.9	<.001^b^	69.6	29.9
KMW	(mm)	3.21	1.38	3.39	1.23	0.42	0.49	.000^b^	2.35	1.76
Bone loss	(mm)	0.081	0.40	0.18	0.66	0.44	0.87	<.001^b^	0.27	0.74

**Notes.**

SD standard deviation PCR plaque control record KMW keratinized mucosa width

a Chi square test was used to compare categorical variables.

b Kruskal–Wallis test was used to compare continuous variables.

*P*-value of < .05 was considered to indicate statistical significance.

Demographic characteristics known to be risk factors for the development of peri-implantitis (age, sex, follow-up period, history of periodontitis, diabetes, smoking, plaque control record, and clinician-associated factors) were used for PS analysis, because large differences were adjusted for in each group. In the final analysis, unstandardized regression coefficients (B), 95% confidence intervals (CI), and *P*-values were estimated with reference to group C. PS analysis showed that both groups A (B = −0.37, 95% CI [−0.49 to −0.24], *P* < 0.01) and B (B = −0.21, 95% CI [−0.30 to −0.12], *P* < 0.01) had significantly lower MBLC than group C (Table 2).

**Table 2:** Comparing the bone loss among three groups.

	**B**	**95% CI**	***P* value**
**Group A**	−0.37	−0.49–0.24	<0.01
**Group B**	−0.21	−0.30–0.12	<0.01
**Group C**	Reference		

**Notes.**

B unstandardized regression coefficients CI confidence interval

## Discussion

Prevention and control of peri-implant disease is crucial in implant therapy. Many factors, such as oral hygiene, periodontal disease, smoking, systemic diseases, implant surface texture, implant site, and prosthesis design, have been proposed as risk factors for peri-implant disease (Aguirre-Zorzano et al., 2015; Gurgel et al., 2017; Staubli et al., 2017; Pjetursson et al., 2012; Marrone et al., 2013; Daubert et al., 2015). In this study, we clarified the correlation between the amount of keratinized mucosa and the bone loss around dental implants and the effectiveness of keratinized mucosa transplantation by PS analysis, which could adjust the difference of related variables. In a cross-sectional study, the lack of keratinized mucosa around the implant was significantly associated with increased plaque accumulation, mucosal recession, interproximal bone resorption greater than 3 mm, and peri-implantitis (Kungsadalpipob et al., 2020) while another cross-sectional study suggested that a thin gingival phenotype and inadequate KMW (<2 mm) could be strong risk indicators of peri-implant disease and pain/discomfort during brushing (Gharpure et al., 2021). In particular, a meta-analysis identified that sites with a wider range of keratinized mucosa had a statistically significant advantage in terms of plaque score, modified gingival index, mucosal recession, and attachment loss (Wennstrom & Derks, 2012). A 10-year cross-sectional study after implant placement showed that a lack of keratinized mucosa was associated with higher plaque score, even in patients with adequate oral hygiene (Roccuzzo, Grasso & Dalmasso, 2016).

Many studies suggest that lack or narrowing of keratinized mucosa may adversely affect oral hygiene; however, there is limited evidence that this factor increases the risk of peri-implantitis, necessitating further research in the future. Conversely, another cross-sectional study demonstrated that soft tissue transplantation with implant placement using CTG reduced bleeding on probing and pocket depth and incidence of peri-implant mucositis and peri-implantitis and had a beneficial effect in maintaining peri-implant health (Obreja et al., 2021). A systematic review showed that soft tissue transplantation provides better peri-implant environment (Thoma et al., 2018). Specifically, the acquisition of keratinized mucosa using autologous grafts greatly decreased the bleeding index, maintained marginal bone height, and increased mucosal thickness using autologous grafts significantly decreased marginal bone loss.

In contrast, excessive thickness of vertical soft tissue surrounding the implant in patients with a history of periodontitis was reported to adversely affect the health of implant tissue (Zhang et al., 2020). Not surprisingly, periodontopathic bacteria have a high risk of colonizing deep pockets around implants, increasing the likelihood of peri-implant disease. Whether maintenance of soft tissue transplant thickness around implants is important requires further investigation.

This study had some limitations. First, this was a retrospective study, and therefore, various biases could not be completely eliminated. In the future the risk factors should be confirmed by conducting prospective studies on diseases with a relatively high incidence, such as peri-implant diseases. Second, this study did not investigate local plaque deposition. As mentioned above, it is important to investigate these factors as the existence of the keratinized mucosa is suggested to have an effect on oral hygiene.

## Conclusions

The results in this study show the importance of keratinized mucosa in maintaining the peri-implant bone. Our findings also suggest that mucosal transplantation is useful, as opposed to narrowing of the keratinized mucosa.

## Supplemental Information

10.7717/peerj.13598/supp-1 Supplemental Information 1 Original data set for statistical analysis

## Additional Information and Declarations

### Data Availability

The following information was supplied regarding data availability: The raw data are available in the Supplemental File.
